# Inferring the Evolutionary Model of Community-Structuring Traits with Convolutional Kitchen Sinks

**DOI:** 10.1093/sysbio/syae026

**Published:** 2024-05-20

**Authors:** Avery Kruger, Vaishaal Shankar, T Jonathan Davies

**Affiliations:** Department of Botany, University of British Columbia, Vancouver, BC V6T 1Z3, Canada; Machine Learning Research, Apple Inc, Cupertino, CA 95014, USA; Physical Stores, Amazon, San Francisco, CA 94105, USA; Department of Botany, University of British Columbia, Vancouver, BC V6T 1Z3, Canada

**Keywords:** Convolutional Kitchen Sinks, community phylogenetics, early burst model, environmental filtering, late burst model, phylogenetic clustering

## Abstract

When communities are assembled through processes such as filtering or limiting similarity acting on phylogenetically conserved traits, the evolutionary signature of those traits may be reflected in patterns of community membership. We show how the model of trait evolution underlying community-structuring traits can be inferred from community membership data using both a variation of a traditional eco-phylogenetic metric—the mean pairwise phylogenetic distance (MPD) between taxa—and a recent machine learning tool, Convolutional Kitchen Sinks (CKS). Both methods perform well across a range of phylogenetically informative evolutionary models, but CKS outperforms MPD as tree size increases. We demonstrate CKS by inferring the evolutionary history of freeze tolerance in angiosperms. Our analysis is consistent with a late burst model, suggesting freeze tolerance evolved recently. We suggest that multiple data types that are ordered on phylogenies, such as trait values, species interactions, or community presence/absence, are good candidates for CKS modeling because the generative models produce structured differences between neighboring points that CKS is well-suited for. We introduce the R package *kitchen* to perform CKS for generic application of the technique.

Phylogenetic approaches in ecology, which incorporate evolutionary history in analyses of ecological patterns, are now widespread (Davies 2021), facilitated by the increasing availability of more inclusive and better resolved phylogenetic trees (Cadotte and Davies 2016). Because species are products of evolutionary history, phylogeny may provide insights into the mechanisms structuring community assembly by capturing information on the expression of conserved traits. In an influential paper, Webb et al. (2002; see also Webb 2000) presented a framework for disentangling the drivers of community assembly through their phylogeny. Following the ecophylogenetic framework presented by Webb et al. (2002), the two primary mechanisms of community assembly that have attracted the most attention are filtering and limiting similarity (although various extensions have been proposed, see, e.g. Cavender-Bares et al. 2009).

Under a filtering process, membership of a community is restricted to those species expressing a particular set of traits or trait values. Freeze or drought tolerance are two clear examples of traits that might be strongly filtered, with species lacking some minimum trait threshold excluded from the community (Chase 2007; Pescador et al. 2016, 2018). If filtered traits are phylogenetically conserved, ecophylogenetic theory suggests that filtering should result in a community clustered on its phylogeny; community members are more closely related to each other than expected by chance because close relatives will tend to share similar trait values. In contrast, under a model of limiting similarity, species cannot co-occur when their traits are too similar. This can be manifested through competitive exclusion. Limiting similarity will result in overdispersion of a community on a phylogeny when traits are evolutionarily conserved, and thus community members will appear more distantly related than expected from a random sample of the species pool.

Assuming a given model of trait evolution, we may also be able to infer the process structuring community assembly simply from its phylogeny and observed trait values of community members without needing to reference the wider species pool (Davies et al. 2012; Davies 2021). However, inference about community assembly processes is complicated by a number of assumptions about the relationship between phylogeny and trait values and that between trait values and direction of interaction (i.e. competition versus mutualism) (Gerhold et al. 2015). Irrespective of the community structuring process, phylogenetic patterns in community membership may retain the imprint of the ecological history, macroevolution, and biogeography of clades, as well as the feedbacks between them that cascade to influence smaller scale processes shaping patterns of coexistence, if we can query them appropriately. One useful starting point is to identify macroevolutionary patterns controlling habitat or biome membership across different lineages (c.f. habitat lineage-pools in Gerhold et al. 2015).

Community structure might additionally feed back to shape trait evolution (Harmon et al. 2019) such that the community phylogeny may contain information about the evolutionary history of those traits (Pearse et al. 2019). For example, previous work has found associations between local community phylogenetic structure and evolutionary rates (Pearse et al. 2019), supporting a link between community assembly and trait evolution. If the process structuring a community is known, it is further possible that community phylogenetic structure might provide insight into the evolutionary trajectory of the traits on which the community was structured, even if trait values are unobserved. Here, we define a community broadly as a species assemblage structured by a given process, and we can thus extend this principle to explore the macroevolutionary patterns of habitat membership across a phylogeny.

Different evolutionary histories can be described as effective trees—transformations of the true tree on which traits evolve (Fig. 1). For instance, traits evolved under an adaptive radiation or early burst (EB) model will appear to have evolved on a tree that is more “stemmy” than the true tree, that is, with longer stem branches subtending clades. In contrast, traits that have evolved under a late burst model (identical in form to an Ornstein–Uhlenbeck (OU) process; Uyeda et al. 2015) will appear to have much of their variation evolved at the tips, returning a “tippy” tree with long branches toward the tips of the phylogeny. These tree transformations can be represented by parameters that reflect the modification of the underlying true phylogeny with branch lengths proportional to time. Here, we present 2 methods to capture information on community structure and infer the effective trees of macroevolution controlling habitat membership.

**Figure 1:**
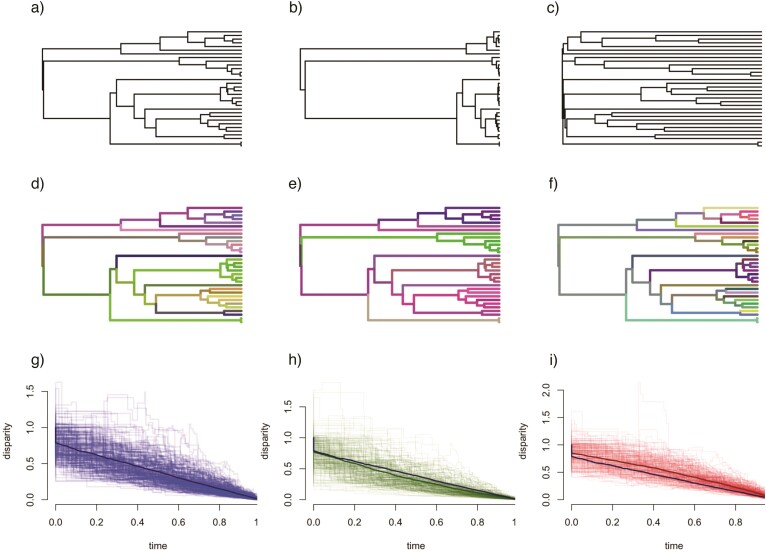
Different modes of evolution can be illustrated as effective trees on which traits evolve. In the top row are different effective trees of trait evolution, representing a) a BM model, b) an EB model, and c) a constrained or late burst evolutionary model of trait change. In the middle row, trees d, e, and f show examples of corresponding traits evolved on the effective trees, but displayed on the true tree, where branch lengths are proportional to time; shades reflect the value of three traits projected in colorspace. Compared with a null model of BM a) and traits evolved on it d), an EB model b) has more trait evolution earlier on, resulting in more similar traits values at the tips e). In contrast, the late burst or constrained model of evolution c) has traits evolve more toward the tips of the tree, resulting in larger trait differences between closely related species f). These different evolutionary models can be captured in disparity through time plots (bottom row, g)–i), illustrated using 200 simulations of trait evolution for each evolutionary model), displaying how trait variance within clades decreases with respect to trait variance among clades. Means of the simulations are illustrated with dark, thick lines; the mean line of BM g) is replicated in h) and i) for comparison. On average, BM results in a linear decrease in disparity through time g), while in the EB model, there is greater between-clade disparity early in the evolution of the phylogeny h), and in constrained or late burst models, there is greater within-clade disparity until late in the evolution of the phylogeny i).

First, we describe community membership using measures of the phylogenetic distance separating species (Webb 2000). We focus here on the mean pairwise phylogenetic distance (MPD) between species pairs in a community, a well-established metric in community phylogenetics often used to test the degree of clustering or dispersion of a community. MPD is a useful metric because it includes information about all branches on a phylogeny, and its standardized version removes its relationship with species richness. In contrast, phylogenetic diversity, the sum of branch lengths connecting a set of taxa, scales closely with richness, and mean nearest taxon distance (MNTD), another common ecophylogenetic metric, only considers branches at the tips of a phylogeny and thus does not capture information on evolutionary relationships deeper in time (Swenson 2011; Kellar et al. 2015; Mazel et al. 2016). Observed MPD can be compared to null expectations generated from drawing the same number of species at random from the species pool (see Kembel 2009); communities that are filtered on phylogenetically conserved traits will tend to have lower MPD than null expectations, indicating phylogenetic clustering. Branch-based metrics, such as MPD, are by definition affected by tree edge (branch) lengths, so they are sensitive to the transformation of tree branch lengths that occurs when known phylogenies are rescaled to represent effective trees describing alternative models of trait evolution (e.g. EB transformation).

We explore a novel application of MPD, calculating the metric across a gradient of tree transformations–effective phylogenies–from extremely stemmy to tippy. Because the series of MPD values across this gradient will capture the contribution of branches at various depths to community structuring, it may allow us to infer the shape of the effective phylogenetic tree that generated the trait data that structured the community. We predict that when a community is structured by traits evolved according to a given evolutionary model, there will be a relationship between the parameter describing that model and the set of MPD values calculated on transformations over a series of parameters, which we refer to as the MPD curve. To apply this approach, it is necessary to first simulate data by evolving traits for the specified evolutionary model across a range of evolutionary parameter values, simulate community assembly, and then train a model relating the evolutionary parameter to the MPD curve of each simulation. Inferences drawn from this approach are, of course, predicated on the technicalities of the simulations. An evolutionary parameter estimated from empirical data suggests that, assuming the simulated community assembly process acted on traits evolved on the known phylogeny, the traits that structured the community evolved in a pattern most consistent with the given underlying evolutionary model. One could in practice find equivalent parameters between different tree transformation models when there is a monotonic relationship between parameter and degree of phylogenetic conservation, as for EB and delta transformations.

Second, we use a novel implementation of a recent machine learning technique called Convolutional Kitchen Sinks (CKS, also known as convolutional random features), for which we provide an R package, **kitchen**. The kitchen sink approximates a kernel—the underlying function that maps an input to an output—using random projections or convolutions (see “Methods” section). Applying a non-linear function to the convolutional random features allows for the approximation of non-linear or non-polynomial functions or relationships between data (x) and an associated value (y), and the error of approximation can be determined mathematically (Hashemi et al. 2023). One key property of CKS is that it allows for the consideration of local relationships between data points. CKS calculates features on a series of windows in a data set; the partial overlap of these windows and exclusion of distant data points in each window allows these features to describe structured patterns in the data. When an observation of species data is ordered in the manner of tips on a phylogeny, the similarity between phylogenetically conserved values of proximal data will reflect the underlying evolutionary distance. CKS can, therefore, take advantage of the patterns in such organized data to estimate the degree of phylogenetic conservation. We can easily extend this to community membership. If species assemblages are structured on (organized by) phylogenetically conserved traits, community membership will contain implicit information on the phylogenetic structure of those traits. For example, under an EB model of evolution, members of young clades will have similar trait values, and filtering community membership by trait values will result in patterns of strongly clustered presence or absence. Conversely, observing tight clusters of presence would suggest an EB evolutionary model given filtered community assembly. These patterns make it possible to estimate the parameter values describing the evolutionary mode of trait change as a function of presence–absence data transformed by CKS.

CKS is essentially a simplification of a convolutional neural network (CNN), using many random features on a single layer rather than learning features across multiple layers (Cho 2012). Like CKS, CNNs use a series of windows to learn features between nearby data. However, CNNs learn the weights of their features over a series of layers. In contrast, CKS uses a set of random features. Random features tend to perform much faster than more complex neural networks, for example, Dempster et al. (2020), since learning features through backpropagation or other algorithms is computationally expensive, but often perform comparably (Hashemi et al. 2023). Here, we apply CKS to a regression problem to predict continuous measures, but CKS features can also be used to solve classification problems.

For both methods we describe, we suggest that it may be possible to learn the evolutionary history of the traits on which a community has been structured even without information on the traits themselves. Underlying our interpretation of these methods is the assumption that evolutionary distances provide reasonable proxies for trait differences (Cadotte et al. 2017). Such assumptions are not always justified, even for traits evolving under strict Brownian motion (BM) (Letten and Cornwell 2015), which is frequently used to quantify the strength of trait phylogenetic conservatism (Blomberg et al. 2003). Nonetheless, phylogenetic distance can provide a good proxy for species differences when integrating over multiple traits (Cadotte et al. 2017), such as might reasonably characterize a species’ position in Hutchinsonian niche space.

In this paper, we simulate trait evolution and community assembly on simulated trees under a spectrum of evolutionary models from EB to late burst, described by an evolutionary parameter that represents the effective tree transformation. We evaluate how well kitchen sink and MPD models predict the true evolutionary parameter for these simulated communities, and show that both are effective in simulated conditions. Finally, we apply our methods to an empirical data set that describes freeze tolerance in angiosperms (Zanne et al. 2014). Our results are consistent with a late burst evolutionary model of freeze tolerance, with most evolution occurring toward the tips of the phylogeny.

## Materials and Methods

We present a method to estimate a rate parameter describing the evolutionary history of community-structuring traits solely based on species presence/absence data. Using simulations, we first explored how the evolutionary signature of traits on which species are filtered into communities is reflected in the phylogenetic structure of the resulting communities, and then evaluated whether it is possible to reconstruct the underlying model of trait evolution using only the species phylogeny and information on community membership.

### Simulated Communities

We used simulated trait-filtered communities in which the true model of evolution was known, and contrast alternative evolutionary models. In this method, we assume a process of community assembly and infer the evolutionary model most consistent with the community observed. In contrast to other ecophylogenetic approaches, our results do not provide information on the likelihood of one assembly process as opposed to another; they only suggest an evolutionary parameter given the assumed model of assembly, not unlike making predictions with any statistically fit model.

We generated phylogenies of different sizes in the R package *geiger* using a birth–death process (b=0.8, d=0.2) (Pennell et al. 2014). We then simulated the independent evolution of continuous traits under different modes of evolution (detailed below), including a simple random walk via BM, and a spectrum of rate variable models. Trait models were applied by transforming the original tree with the rescale function in *geiger* (Pennell et al. 2014) using selected transformation parameters (evolutionary parameters). Traits were then simulated on the transformed trees using the function fastBM from the *phytools* R package (Revell 2012). Because there can be large variation in the phylogenetic signal in trait values even when evolved under a common model, we characterized species trait similarities in multidimensional trait space. We used 10 traits per simulation; exploratory analyses showed that models continue to improve with increasing trait count at an exponentially decreasing rate (Supplementary Fig. S1). We evolved traits independently on one effective tree; see Sensitivity of CKS to Alternative Models below for exploration of alternatives.

We generated 6 phylogenies of varying sizes (48, 64, 128, 256, 512, and 1024 species). For each tree, we simulated 2000 communities, which were separated into 1200 simulations used to train models and 800 simulations used to validate models. In each simulation, we evolved 10 independent traits on an effective tree that had been transformed by a given evolutionary model/parameter. We created filtered communities by selecting the 30 species that had traits closest in Euclidean space to a 10-trait optimum chosen from an initial arbitrarily selected species, producing presence–absence data across the phylogeny.

We here use the EB tree transform (Pagel 1999) as illustration. The EB transformation is an exponential transformation of branch lengths, specified by parameter *a*, such that rate of evolution increases or decreases exponentially with time ($r\,a\,t\,e_{t}=r\,a\,t\,e_{0}\times e^{a\,t}$) (Pennell et al. 2014). Parameter values close to 0 result in a BM or random walk-like model of evolution, while negative parameter values represent an EB model in which evolution slows over time, positive parameter values create a late burst model in which evolutionary rates increase with time, and large positive parameter values approach a white noise model where species traits are effectively random. We selected an EB transformation parameter, *a*, for each simulation from a normal distribution with mean 0 and standard deviation sd=5/tree depth. We adjusted the standard deviation between trees because our larger trees are deeper, and thus convergence on white noise or uniformity was reached at smaller absolute parameter values due to the exponential nature of the EB transformation. More complex evolutionary models could be explored, for example, by allowing rates to vary among clades, if there are *a priori* expectations that such models might capture the underlying process of trait evolution.

In simulated data, we know the generative model and our predictions are, therefore, directly interpretable with reference to this underlying model. However, in empirical data, the true generative model cannot be known. Empirical estimates based on simulated data must therefore be interpreted as predictions assuming the generative model used in simulations. Nonetheless, predictions on empirical data may still be broadly interpretable (e.g. supporting an early vs. late burst model), and models based on different generative models will likely produce predictions that lead to the same qualitative interpretations, as we explore below with the delta transformation.

### Phylogenetic Structure Metrics

For each simulated community, we characterized the community phylogenetic structure using the standardized mean pairwise phylogenetic distances (MPD) as a metric of phylogenetic dispersion, which describes the average relatedness of species in a set, and is often compared to a null expectation of a random assembly from the species pool (Kembel 2009). We then generated a series of effective trees by applying EB transformations to the original tree, scaled to time, from a=−3 to a=3. We stepped *a* by increments of 0.1, and by increments of 0.05 between −1 and 1, creating a gradient of 81 transformed trees from stemmy (EB-like, with lengthened branches toward the base and shortened branches toward the tips) to tippy (late burst-like, with shortened branches toward the base and longer branches toward the tips). We calculated MPD on each of the EB-transformed trees to generate a series of MPD values matched to *a* transformations—the MPD curve. For each of the 6 simulated trees, we used the training data to model the known values of *a* on which the simulated communities were evolved as a linear function of the matching MPD curves. We used the trained models to predict the *a* values of the validation data and then regressed the known *a* values of the validation data on the predicted values to test the predictive ability of the models.

We also explored how the implicit evolutionary model underlying the MPD curve affected our results. We produced MPD curves with a delta transformation, an alternate evolutionary model distinct from the EB transformation used to simulate communities. We again created a series of trees on a gradient from stemmy to tippy, using delta transformations rather than EB transformations. We then trained linear models relating the true EB parameter to the MPD curves, as above, and examined the predictive power of the alternative model curves.

### The Convolutional Kitchen Sink

The relationship between points (species) in presence–absence data may contain information about the underlying phylogeny if presence is determined by trait values and the species are ordered in the manner of tips on the phylogeny (i.e. preserving monophyly of clades). To query this evolutionary signature, we applied a powerful machine learning technique, CKS, which can efficiently capture structured nonlinear relationships between variables and has been shown to be effective across different scientific domains (Rahimi and Recht 2008; Morrow et al. 2017). We developed the **kitchen** package to apply CKS to ecological and evolutionary data in R.

CKS can be interpreted as training the last layer of a randomly weighted neural network (Cho 2012). If a vector of species presence/absence $\vec{x}$ is the output of a function in which species are assembled into a community based on traits evolved on an underlying effective tree T described by parameter a, then by training a linear model on data transformed by CKS, we can learn this function, allowing for the estimation of a given $\vec{x}$. We introduce the R function **kitchen_sink()** to perform the technique easily.

**Figure 2:**
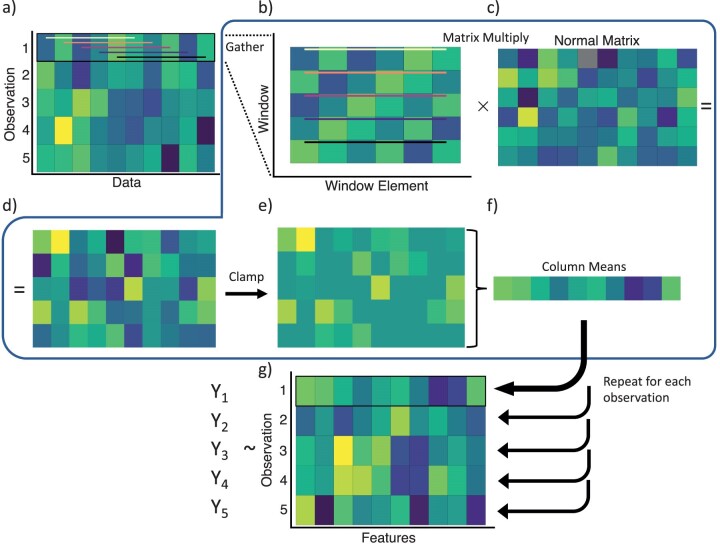
An illustration of the steps to perform a CKS projection. a) Begin with a matrix of data, where rows represent a single observation, such as a vector of species presence–absences or abundances in a community. For each observation, the following is done: b) All windows of a chosen size w (w=6 in this illustration) are gathered into a single matrix; colored lines show how each window matches with data in a). Next, this matrix is multiplied by c) a normal matrix of dimensions w×f, where f is the desired feature count ( f=10 in this illustration). This matrix should remain the same between observations. d) Matrix multiplication results in random features. e) Negative values are clamped to 0. f) The mean of each column is then calculated, producing an average across windows for each feature, the final product for one original observation. Steps a)–f) are repeated for each observation using the same normal matrix, and the features are gathered into g) a final matrix where rows correspond to the original observations and columns represent convolutional random features. Known values associated with the data can then be modeled as a function of these random features with a simple ridge regression. Trained models can make predictions on data transformed with the original normal matrix.

CKS depend on 2 hyperparameters, window size and feature count. Window size describes how many consecutive data points should be considered at a time, allowing for local structure in data. For example, using windows of size 6, a model would include relationships between points within each of a series of overlapping windows consisting of data points 1–6, 2–7, 3–8, and so on, but would not include relationships between points further apart (Fig. 2a). From a data perspective, optimal window size could be used to interpret the scale of local structure in the data; an optimal model using small window sizes suggests that relationships between nearby points are important in determining an outcome. A feature describes a random weighting of all data in a window passed through a nonlinear function. The number of features at which information no longer is gained could be interpreted as the complexity of the relationship between the data and the corresponding variable of interest, though this is tempered by the fact that only some of the random features are ultimately useful to the model (e.g. linear model) that is used.

To pass a matrix of data through a convolutional kitchen sink of window size w and feature count f, all windows in an observation (i.e. row $\vec{x}$) are collected into a matrix, resulting in a matrix of dimensions (l⁢e⁢n⁢g⁢t⁢h⁢(x)−w+1)×w, where each row is a window of data (Fig. 2b). This matrix is then multiplied by a random normal matrix of mean 0, standard deviation 1, and dimensions w×f (Fig. 2c–d). The compute time for this scales linearly with (l⁢e⁢n⁢g⁢t⁢h⁢(x)−w+1)×w×f (Supplementary Fig. S2). The product must then be passed through a nonlinear activation function because any combination of linear functions can only result in a linear function, while piecewise linear functions or other nonlinear functions can approximate arbitrary nonlinear functions. We use a rectifier or RelU function that sets negative values to 0 (Fig. 2e), which can be performed in R with our function **clamp()**. Each feature (i.e. column) of the nonlinearized matrix is then averaged across all windows (i.e. across rows) (Fig. 2f). These steps are repeated for each observation (row) in the original data, using the same normal matrix. This scales the compute time of CKS with the number of observations n. Finally, the resulting vectors for all observations are put into the rows of a matrix, with dimensions n×f, where each row is a set of features corresponding to the rows of original data and each datum represents a feature describing the nonlinearized random weighting of all original values within defined windows (Fig. 2g). **kitchen_sink()** performs all of these steps when provided with a matrix of data (where rows correspond to observations) and a normal matrix, which can be generated with our function **make_norms()**.

The output of **kitchen_sink()** is a matrix of random features with rows corresponding to the rows of the input data. A simple linear regression or ridge regression can fit a corresponding vector to the rows of the data (e.g. a vector of parameters that generated or are associated with the observations). Linear regressions are liable to overfit data as the number of features increases; in contrast, ridge regression applies a penalty to increasing data, preventing overfitting, and making feature size simply a choice of how much computational power to use until more features no longer provide new information. Predictions can then be made from new data by passing these data through a kitchen sink using the same normal matrix applied to the training data, and predicting on transformed data with the trained model. We provide the function **kitchen_predict()** to predict values from observations given a training data set.

The computational time to perform CKS and train a model on the output depends on the length of an observation l, the number of observations n, window size w, and feature count f. The time complexity to calculate the random features of all the observations is $O\,\left(l\,e\,n\,g\,t\,h\,\left(\vec{x}\right)\times w\times f\times n\right)$ (Supplementary Fig. S2). Training a model on the random features has an approximate time complexity $O\,\left(f^{2}\right)$ (Supplementary Fig. S3). As dimensions increase, the random feature calculation takes an increasing proportion of total compute time, so the total time complexity is $O\,\left(l\,e\,n\,g\,t\,h\,\left(\vec{x}\right)\times w\times f\times n\right)$. This is equivalent to the time complexity of one gradient evaluation of a neural network, so CKS is computationally faster than neural networks that must perform multiple gradient evaluations.

We applied **kitchen_sink()** to our simulated communities of presence–absence data. We use an input matrix where each row (observation) corresponds to the outcome of a single simulation, consisting of presence and absence data represented as 1s and 0s respectively. Windows include presence and absence of species that have been organized close together such that adjacency reflects phylogenetic relationships in the observation due to their phylogenetic relationships; the series of all windows, therefore, includes and excludes various clades and outgroups. The output of our CKS is a matrix in which each row represents a simulation and each column represents a random feature that describes the relationships between species presences and absences within a window, averaged across windows.

To optimize our hyperparameters, we tested the performance of models trained on CKS outputs across a range of feature and window sizes. We produced models for each CKS by regressing the true underlying *a* parameters on the CKS outputs, using ridge regression to avoid overfitting. To validate each model, we then examined how models trained on the training data performed on the validation data using the function **kitchen_sweep()**, which models the true *a* parameters as a function of the predicted *a* parameters for each kitchen sink model and compares performance with adjusted *R^2^* values. Outlier predictions beyond the range of the training data were clamped to the maximum or minimum of the training data.

### Sensitivity of CKS to Alternative Models

To explore sensitivity to the community structuring process, we also tested the performance of CKS on alternative community assembly processes.

First, we examined whether CKS perform well when communities were assembled probabilistically rather than deterministically. We evolved a 128-species phylogeny with a birth rate of 0.8 and death rate of 0.2. We chose 7 different exponential parameters to produce probabilistic assembly scenarios ranging from near-deterministic to near-random and performed 4000 simulations for each. In each simulation, an EB parameter was chosen from a normal distribution (μ=0,σ=1); we truncated parameters at different positive values depending on the exponential parameter to avoid probabilities that were too small. We transformed the phylogeny with each EB parameter and then evolved 10 traits on the transformed tree. For each simulation, a community of 32 species was assembled by probabilistically choosing species based on their distance in trait space from an optimum (set to the mean of each trait across all species), with probability $e^{\left(-d\,i\,s\,t\,a\,n\,c\,e\times e\,x\,p\,o\,n\,e\,n\,t\,i\,a\,l.p\,a\,r\,a\,m\,e\,t\,e\,r\right)}$. A CKS model describing the relationship between the EB parameter and assembled community was trained on 3000 simulations. We then predicted the EB parameter of the remaining 1000 simulations and calculated the $R^{2}$ of the fit between prediction and known value of the EB parameter.

Next, we examined whether independence of traits affected inference by comparing CKS performance on communities assembled on independent and covarying traits. We evolved a 128-species phylogeny with a birth rate of 0.8 and death rate of 0.2. We then performed 4000 simulations each for the independent and covarying simulations. In each simulation, an early burst (EB) parameter was chosen from a normal distribution (μ=0,σ=1). The phylogeny was transformed by the EB parameter and traits were evolved on the transformed tree. For the independently evolved simulations, 10 traits were evolved with zero predetermined covariance. For the covarying traits simulations, 10 traits were evolved with perfect covariance (i.e. a species had 10 traits of the same value). After trait evolution, a community of 64 species was assembled by choosing species with likelihood $e\,x\,p\,\left(-0.5\times d_{i}\right)$, where $d_{i}$ was the Euclidean distance of species i from an optimum (chosen to be the mean value of each trait across all species), and 0.5 was an intermediate value of the exponential rate parameter λ. Models describing the relationship between the EB parameter and assembled community were trained on 3000 simulations. We then predicted the EB parameter of the remaining 1000 simulations and plotted the predictions against known EB parameters used to generate each simulation.

We also tested whether we could recover the mean or maximum of a set of evolutionary parameters (*a* values) if communities were assembled on multiple traits that evolved on unique effective trees. We examined 2 scenarios, a 2-*a* scenario and a 5-*a* scenario. We evolved a 128-species phylogeny with a birth rate of 0.8 and death rate of 0.2 and performed 4000 simulations for each secenario. In each simulation, parameters were chosen from a normal distribution (μ=0,σ=1). In the 2-*a* scenarios, 5 traits were evolved on each of the two *a*-transformed effective phylogenies. In the 5-*a* scenario, 2 traits were evolved on each of the 5 *a*-transformed effective phylogenies. After trait evolution, a community of 32 species was assembled by choosing species with likelihood $e\,x\,p\,\left(-3\times d_{i}\right)$, where $d_{i}$ was the Euclidean distance of species i from an optimum (chosen to be the mean value of each trait across all species), and 3 was a high value of the exponential rate parameter λ (similar to deterministic assembly). Models describing the relationship between the mean and max of the EB parameters and the assembled community were trained on 3000 simulations. We then predicted the mean and max EB parameter of the remaining 1000 simulations and plotted the predictions against known values used to generate each simulation.

Finally, we examined whether CKS and MPD models could work on communities assembled by limiting similarity rather than filtering. We evolved a 128-species phylogeny with a birth rate of 0.8 and death rate of 0.2 and performed 2000 simulations. In each simulation, an EB parameter was chosen from a normal distribution (μ=0,σ=0.5). The phylogeny was transformed by the EB parameter, and then 10 traits were evolved on the transformed tree. A community of 32 species was assembled by sequentially removing the species with the smallest Euclidean distance in trait space from all the other species until 32 species remained. CKS and MPD models fitting the relationship between EB parameter and assembled community were trained on 1350 simulations. We then predicted the EB parameter of the remaining 650 simulations and calculated the $R^{2}$ of the fit between prediction and known value of the EB parameter.

### Evolution of Freeze Tolerance in Angiosperms

To illustrate our CKS approach, we applied the kitchen sink method to a 9849-species tree of angiosperms with information on freeze exposure (Zanne et al. 2014). We first simulated 5000 filtered communities of size *n* = 4353 (the number of species classified as freezing-exposed in the original data). In each simulation, we used an EB tree transformation with *a* selected from a normal distribution of mean 0 and standard deviation 0.08, clamped to between −0.2 and 0.2, and evolved 10 traits along the branches of this transformed tree. The standard deviation and range of *a* were truncated relative to our previous simulations because EB transformations of our 243-myr old angiosperm tree with large positive or negative parameter values quickly lead to indistinguishable star-like trees (with most evolution occurring at the tips) or stick-like trees (where most evolution occurs on the deepest edges), respectively; we thus constrained our analysis to focus on evolutionary parameters that approached but did not greatly exceed phylogenetically informative parameter space. We then randomly selected a species in the tree to represent an optimum value in trait space, and generated a filtered community by selecting the 4353 species minimally distant to this trait optimum in Euclidean space, as described in our simulations above.

We separated our communities into a 4000-simulation training data set and 1000-simulation test data set, and trained kitchen sink models to predict *a* in the training data set, sweeping from window size 4 to 9849 (the size of our data) by factors of 4, and feature count 16–512 by factors of 2. We then applied the trained models to the validation data set to predict the true evolutionary parameter *a* underlying the evolution of filtered traits of each simulated community. We regressed the known *a* values on the predicted values for each combination of window size and feature count, and chose the combination that maximized $R^{2}$ for application to the empirical data. Next, we used our best CKS model to estimate the evolutionary parameter that best described the structure of freeze-tolerant angiosperms on the Zanne et al. (2014) phylogeny. We assigned values of 1 to plants that were exposed to freezing conditions in their range, reflecting freeze tolerance, and 0 to plants unexposed to freezing conditions across their range. We then predicted an *a* parameter using our trained CKS model. To estimate 95% confidence intervals, we performed 100 bootstraps of the best kitchen sink model by retraining the model using the training data sampled with replacement and again estimating the parameter of the true community with the bootstrapped CKS models (Recht et al. 2019).

For comparison, we also used the MPD curve method to predict the same evolutionary parameter. We calculated MPD curves for all simulated communities and the known community of freeze-tolerant angiosperms. We then trained a linear regression of *a* on the MPD curves of the training data and used the model to predict *a* values from the MPD curves of the validation data and the known community.

## Results

### Parameter Estimation

**Figure 3:**
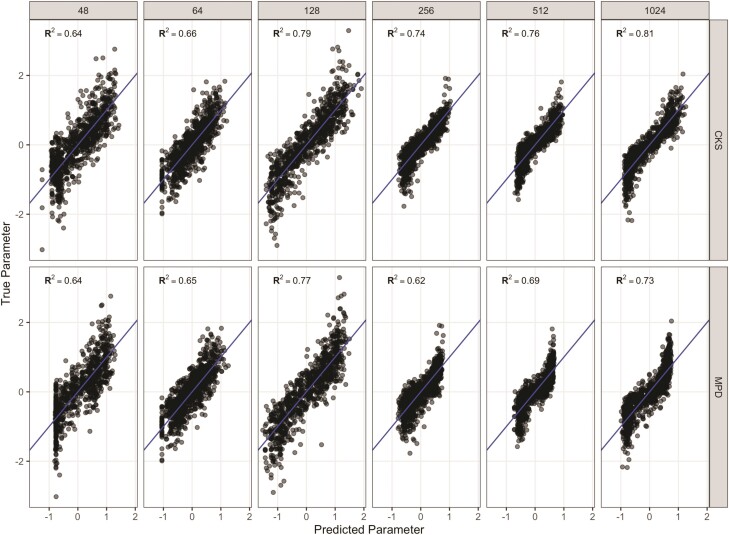
Comparison of performance between models trained on CKS-transformed data (top row) and models trained on MPD curves (bottom row) for trees ranging in size from 48 to 1024 species. Both models performed well across a range of tree sizes, but CKS increasingly outperformed MPD as tree size increased, as indicated by the R^2^ values in each panel. Community size was held constant at *n* = 30 across tree size.

In our exploration of EB models on simulated trees, in which we varied parameter *a*—the exponent to which evolutionary rates accelerate through time—both linear models fitting *a* to MPD curves and our CKS model, trained on presence–absence data from communities structured by trait filtering, were able to predict accurately the true value of *a*. However, CKS increasingly outperformed linear model fits as tree size increased (Fig. 3). Predictive strength of our CKS models also generally increased with tree size (Fig. 3). The parameter prediction curves were typically logit-shaped, with decreasing predictive power at high and low true parameter values (Fig. 3). Reducing the possible range of *a* for simulations on a given tree resulted in a more linear fit, as non-predictive tails for large values pulled other predictions away from the 1:1 slope.

Models trained on delta-based MPD curves performed similarly to those trained on EB-based curves (Supplementary Fig. S4), although the location of the curve minima on the delta-based curves did not correspond linearly with predicted values. Both tree transformations we explored, delta and *a*, are able to create stemmy and tippy trees, allowing the MPD curve to describe how the community structure is clustered across the depth of the tree. Alternative transformations, such as lambda, which is commonly used to quantify phylogenetic signal (Pagel 1999), do not capture this same information. Nonetheless, when using MPD curves, it may be valuable to explore alternative transformations to determine if any are more predictive, for example, due to the unique ways they may isolate information on internal branches within complex tree topologies.

### Sensitivity of CKS to Alternative Models

**Figure 4:**
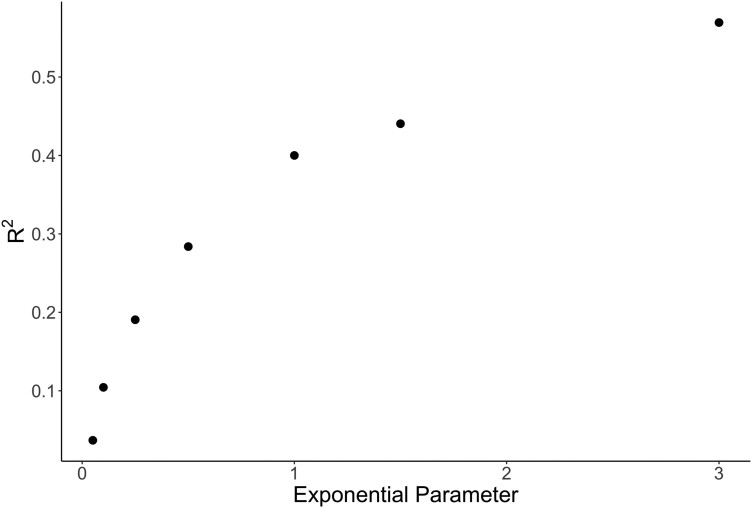
A scatter plot of best R^2^ performance for CKS models trained and tested on different degrees of probabilistic assembly. Increasing exponential parameters approached deterministic assembly, while decreasing exponential parameters approached random assembly.

CKS worked effectively on communities assembled probabilistically, depending on the exponential rate parameter λ. At high λ, probabilistic assembly resembled deterministic assembly and power of inference was high, whereas low λ produced random assembly and low ability to infer evolutionary parameters (Fig. 4).

**Figure 5:**
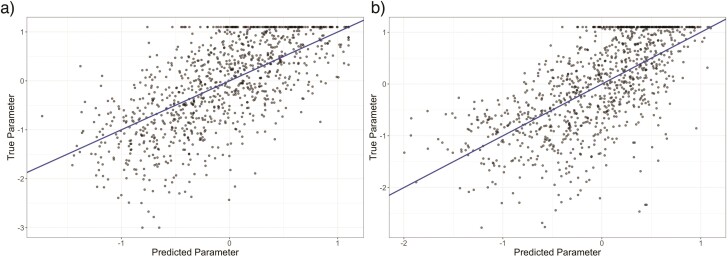
Scatter plots showing the performance of CKS models trained and tested on communities assembled on a) independently evolved traits and b) completely covarying traits. The predictive power of the 2 models did not appear qualitatively different; intermediate modes of covariance produced qualitatively identical patterns.

The predictive power of models based on independent and covarying traits did not appear qualitatively different (Fig. 5). The independent trait model achieved an *R^2^* of 0.472, while the covarying trait model achieved an *R^2^*of 0.435. We present these 2 models as the extremes of trait covariance, but we also explored intermediate modes of covariance and found qualitatively identical patterns.

**Figure 6:**
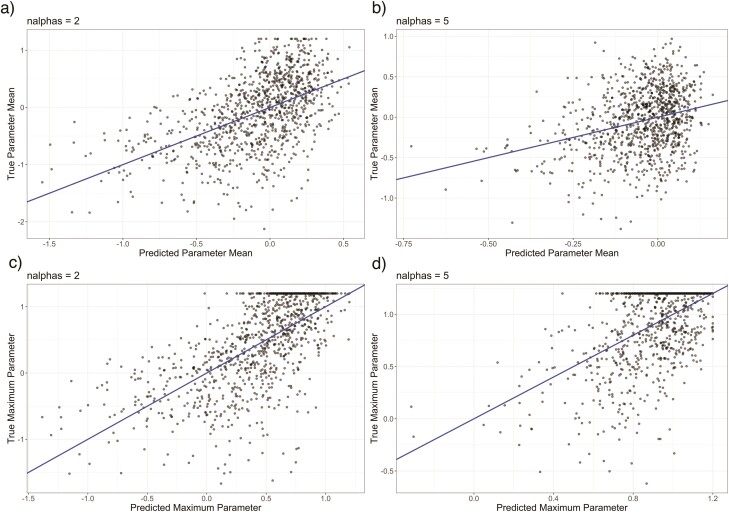
Scatter plots showing the performance of CKS models trained and tested on communities assembled on traits evolved on differing numbers of unique effective trees. Top row shows performance of predicting the mean of a) 2 and b) 5 independent *a* parameters describing effective trees. Bottom row shows performance of predicting the maximum value of c) 2 and d) 5 independent parameters describing effective trees. Performance on recovering the mean declined as the number of *a* values increased (a and b). Our ability to recover the maximum of the *a* values was better c and d), but still poor compared to scenarios where all traits were evolved on a single effective tree (e.g. Supplementary Fig. 1).

When there were independent evolutionary parameters governing the traits communities were assembled on, models were not able to effectively recover the mean of the *a* values, with decreasing power as the number of *a* values increased (Fig. 6, Supplementary Fig. 5). In contrast, our ability to recover the maximum of the values was better, but still poor compared to scenarios where all traits were evolved on a single effective tree (e.g. Supplementary Fig. 1).

**Figure 7:**
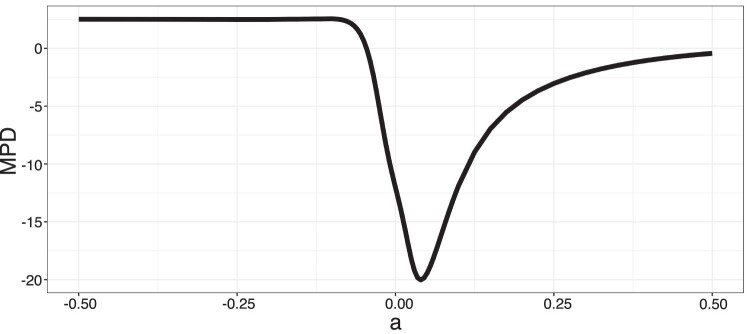
The MPD curve of the known community of freeze tolerant plants. The *a* value maximizing clustering (minimum MPD) is indicative of the tree transformation under which the community structuring traits evolved; however, the linear model trained on MPD curves of simulated communities predicted an *a* of 0.040, somewhat lower than our CKS model.

We found that both MPD and CKS methods were also able to accurately predict evolutionary parameters structuring community assembly by limiting similarity (Supplementary Figs. S6 and S7). The slope of the true parameter over the predicted parameter was close to 1 for both methods and *R^2^*>0.5. The extremes of the predicted parameters were not asymptotic, indicating the range of true parameters used during simulation did not include phylogenetically noninformative parameter space (effective trees were neither stick-like or rake-like). On visual inspection, we found that each prediction by MPD methods corresponded closely to a single feature, the location of the curve minimum, which is the transformation at which a community is maximally clustered.

### Evolution of Freeze Tolerance

Assuming the tree topology from Zanne et al. (2014) and simulated communities filtered on trait values evolved on EB transformations, all kitchen sink models outperformed a simple linear regression on species presence–absence (all kitchen sink models adjusted R^2^>0.20, versus linear regression adjusted R^2^ = 0). R^2^ increased with feature count, but tended to saturate at around feature count = 256. High performance was reached quickly with small window (w) sizes (R^2^>0.87 when w≤1024). Performance decreased slightly at w = 4096 while computing time greatly increased. Performance was worst with w = 9849, the dimension of the full data set.

**Figure 8:**
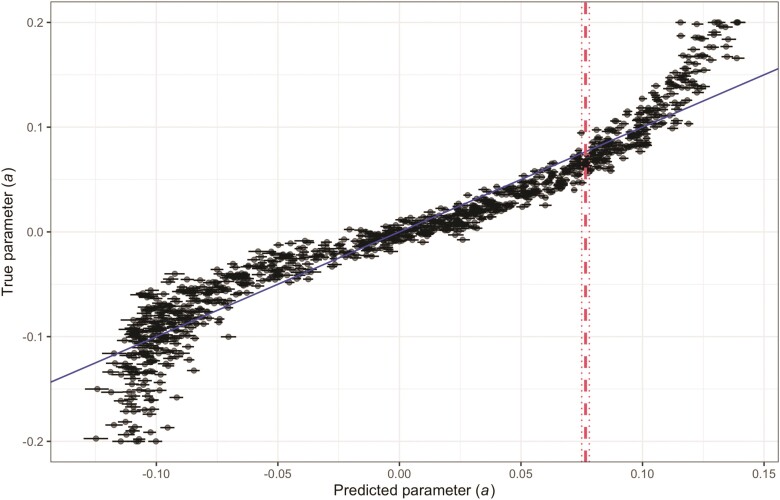
Scatterplot showing the relationship between the true value of the *a* parameter from 1000 validation simulations on the Zanne et al. tree of angiosperms and their mean predicted values across 100 models trained on CKS of bootstraps of training data using 512 features and a window size of 256; horizontal bars represent 95% confidence intervals. The vertical dashed line represents the mean of 100 predictions of the parameter governing evolution of the community-structuring trait (i.e., freeze tolerance) trained on bootstraps of the training data using a CKS models with identical hyperparameters and normal matrices. The dotted lines represent the 95% confidence interval from this bootstrap. The solid diagonal line is an identity line with slope 1.

**Figure 9:**
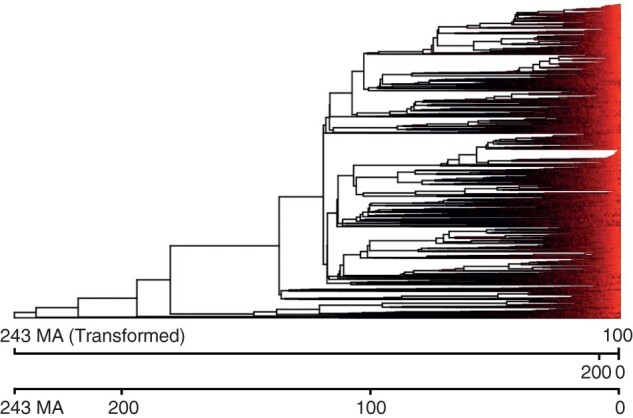
The tree of angiosperms, with branches colored by the predicted rate of evolution of freeze tolerance. Brighter color represents a higher ratio of branch lengths on a tree transformed by the rate parameter to the untransformed branch lengths, indicating a higher rate of evolution of community-structuring traits than expected if rates were constant and evolution is proportional to the true underlying tree. The top scale bar shows the effective age of the tree in terms of community-structuring traits, while the bottom scale bar shows the known scale of the tree.

The CKS model with f=512, w=256 had nominally the best predictive power (R^2^ = 0.925). Applying this model to the empirical data on freeze-tolerant angiosperms using 100 bootstraps of the training data, we predicted an *a* of 0.077 (Fig. 8; 95% CI[0.075, 0.078]). The average prediction across models with w≤1024 was 0.084. These results support a late burst model with rates increasing towards the present (Fig. 9). The linear model trained on MPD curves also supports the late burst model, but returns a predicted *a* of 0.030.

## Discussion

The environmental or biotic filtering of species into communities and regional assemblages is predicted to result in phylogenetic clustering when filtered traits are evolutionarily conserved, and thus the phylogenetic structure of a community may reveal information about the process of community assembly (Webb 2000; Webb et al. 2002). Previous work has explored how phylogenetic metrics of community structure can predict community functional diversity (Swenson 2011) and reveal variation in the phylogenetic depth at which species assemblages are structured (Mazel et al. 2016). It has also been suggested that the phylogenetic structure of species assemblages may capture meaningful information about trait evolution (Davies et al. 2012; Pearse et al. 2019; Davies 2021). Here, we present 2 methods that use the structure of community composition to infer the macro-evolutionary history of community structuring traits.

A variety of phylogenetic structure metrics have been developed (Tucker et al. 2017), of which the most commonly used are the mean pairwise distance (MPD) between species and the MNTD (Kembel 2009). Because the latter only examines distances between closest relatives, it is frequently used to infer phylogenetic structuring toward the tips of the tree, whereas the former metric, which integrates both shallow and deep pairwise distances, is assumed to better capture structure deeper in the tree (Mazel et al. 2016). Comparisons between the strength of clustering indexed by these 2 metrics might thus indicate whether community structuring traits evolved relatively early or late in a clade’s history (Souza et al. 2018; Eme et al. 2020). Here, we present a more refined approach that allows us to address this question directly. We employed a simple tree-transformation to detect the strength of community clustered at different phylogenetic depths. We used a series of EB transformations to rescale the contributions of shallow versus deep branches to MPD, producing “MPD curves,” and show that models trained on MPD curves can predict evolutionary parameters well, especially on small trees (Fig. 3).

Next, we employ a recently developed machine learning tool, CKS, to infer the underlying evolutionary rate parameter describing the evolutionary trajectory of community structuring traits. By simulating trait evolution and community assembly, we produce known communities and associated evolutionary parameters on which we train a suite of kitchen sink models. The models take advantage of the phylogenetic structure underlying the organization of a vector of species’ presence/absence. We show that CKS tends to outperform MPD models and is generally more effective on larger trees (Fig. 3). For the evolutionary model we explore, a rate-variable model in which evolutionary rates can increase or decrease over time, determined by a tree transformation parameter, *a*, predictive strength is greatest at small absolute parameter size (Fig. 7); at large negative parameter values, the tree becomes dominated by deep branches and traits among species within clades become extremely similar, while at large positive values, tips dominate the tree structure, and species traits become effectively independent of phylogeny. Fortunately, phylogenetic signal in traits is common and small *a* values are, therefore, quite likely (Blomberg et al. 2003). We note that while kitchen sink models are not necessarily generalizable across trees (i.e. a model trained on data simulated assuming a given tree topology cannot be applied to data generated under an alternative tree topology), simulations on a given tree can reveal the predictive strength of the modelling approach assuming that tree topology (Fig. 3).

We demonstrate the utility of our approach by applying it to real world data on freeze tolerance in angiosperms. Zanne et al. (2014) used a large phylogeny of land plants and accompanying range and trait data to investigate when traits that enable freeze tolerance, such as deciduousness and smaller vasculature, evolved in relation to the timing of range shifts into environments that experience freezing. This system provides a useful illustration of our approach, which is likely better suited to large-scale regional structuring of species, as we gain statistical power from examining patterns across multiple lineages. We show that in a model in which plants are assembled into freezing-exposed communities or lineage-pools by the filtering out of freeze-intolerant plants, we estimate a small, positive rate parameter on an EB transformation, suggesting a late burst model of evolution of freeze tolerance (Fig. 8). Our results suggest support for freeze tolerance arising later in angiosperm evolution, matching expectations given the predominantly tropical origin of angiosperms (Doyle 2012).

Our approach does not allow us to determine absolute rates of evolution because communities were assembled by relative rather than absolute distance from an optimum (we modeled filtering by selecting n species closest to an optimum, rather than those within some predefined distance, so the simulations were therefore insensitive to the absolute rate of evolution). Our results are, therefore, also consistent with a model of constrained evolution, such as a characterized by an OU process. OU transformations produce trait distributions identical to that of late burst models of accelerating trait evolution (Uyeda et al. 2015). We favor the late-burst interpretation of freeze tolerance evolution because it matches expectations of evolutionary rates accelerating in response to a changing global climate and the emergence of cooler temperatures in the Cenozoic (Schubert et al. 2019b; Shiono et al. 2018). Global temperatures began dropping 50 million years ago (Ma) during the Eocene, and temperate regions expanded during the following Oligocene (34-23 Ma), producing selection for freeze tolerance in these areas (Schubert et al. 2019a). The timing of these events is qualitatively consistent with Figure 9, in which the rate of evolution of freeze tolerance appears to accelerate around 30 Ma.

Our findings supporting a late burst pattern of evolution of freeze tolerance are consistent with previous studies that have found EB patterns of trait evolution to be rare (Blomberg et al. 2003; Harmon et al. 2010). Although a late burst of freeze tolerance aligns with a period of global climate change, a variety of macroevolutionary processes could produce or contribute to a late burst pattern. For example, evolution could be rapid on short time scales but fluctuate and or be constrained, limiting the long-term signal of microevolution in macroevolution (Harmon et al. 2010; Uyeda et al. 2011). Uyeda et al. (2011) suggest that rapid periods of evolution that persist long term might reflect large changes in adaptive zones. Habitat is an obvious candidate for a large-scale adaptive zone, and traits mapping to habitat or niche occupancy may, therefore, provide opportunities to explore how macroevolutionary patterns can be related to ecological shifts. The prevalence and mode of adaptive radiations remain topical questions in ecology, for example, Schluter (2000). The creation or expansion of biomes may form the basis for radiations, so comparing the tempo of evolution of occupancy versus other traits could suggest if phenotypic divergence tends to occur *in situ* after radiation in a biome or preadaptively prior to invasion. The freeze-exposed niche we use is one example of a relatively recent, rapid biome expansion. Others include the expansion of African savanna around 10–15 Ma (Davies et al. 2020) and that of the Nama-Karoo, a grassy south African biome, in response to climatic changes during the last interglacial, including reduced upwelling from the Benguela Current (Urrego et al. 2015).

We present the example of freeze tolerance in angiosperms for illustration only, and we recognize that our global analysis could be misled. For example, the multiple independent origins of freeze tolerance in different clades and locations around the globe means a single rate parameter may not provide accurate representation of tree-wide rates. Greater precision would likely be possible using regional phylogenies or more complex evolutionary models. It would be relatively straightforward to expand our model to explore both extensions. A notable advantage of kitchen sink models is that they can easily be simultaneously trained on multiple parameters, as long as there is appropriate ground-truth data for training. Models can be simply trained on separate sets of parameters using the same CKS output.

Our approach relies on some key assumptions. The underlying model of community assembly we use assumes an evolution of trait “syndromes” rather than any specific individual trait, although we believe this is a reasonable assumption for many ecological filtered species attributes. Because of stochasticity in both trait evolution and community assembly, our estimates are necessarily imprecise, and are likely most useful for evaluating broad patterns and large data sets. Further, we have not evaluated our approach on communities assembled by a mix of processes, and it is likely that a model trained on communities assembled via one process would not perform as well in evaluating a community assembled on a different process. Nonetheless, we believe our method provides an important advance over simple comparisons between different metrics of phylogenetic structure, such as the mean pairwise distance between community members and mean nearest-neighbor pairwise distance, that are assumed more or less sensitive to patterns at different phylogenetic depths (Swenson 2011; Mazel et al. 2016). In addition, we show that CKS also performs well in estimating evolutionary parameters of community structuring traits when communities are structured by limiting similarity, and we are even able to reliably estimate evolutionary parameter values using community simulations generated under alternative (albeit related) evolutionary models.

Finally, we highlight CKS as a useful and computationally light machine learning technique. By using random features, CKS avoids the need to learn features with expensive computation like backpropagation (Hashemi et al. 2023). Time to train and predict varied across scenarios, as compute time of matrix multiplication scales with the product of matrix dimensions (size of phylogeny, window size, and feature size) and linearly with the number of simulations. *kitchen* makes CKS straightforward to apply, and we hope it becomes an accessible technique to both ecologists and evolutionary biologists. Previous applications have included predicting binding sites of transcription factors (Morrow et al. 2017) and land attributes from satellite imagery (Rolf et al. 2021). The technique is particularly useful for large data sets and data that can be simulated, both of which allow for highly trained models. The genomics revolution, for example, may provide many opportunities to apply CKS. We suggest data generated on phylogenies, such as trait values, are also good candidates for CKS because the underlying tree produces structured relationships between neighboring data—local patterns within groups of nearby points that CKS can learn from. Other scenarios in which a generative process results in patterns on tree tips—for example, symbioses characterising microbe–host parasite–host associations, or pollinator–flower interactions—could also make good targets for this technique.

## Supplementary Data

Data available from the Dryad Digital Repository: http://dx.doi.org/10.5061/dryad.zw3r2289q.

## Data Availability

The *kitchen* R package is available at https://github.com/avery-kruger/kitchen. The phylogeny and associated data from Zanne et al. (2014) are available at https://doi.org/10.5061/dryad.63q27.
